# Dissecting the mediating role of inflammatory factors in the interaction between metabolites and sepsis: insights from bidirectional Mendelian randomization

**DOI:** 10.3389/fendo.2024.1377755

**Published:** 2024-08-14

**Authors:** Fangchen Gong, Wenbin Liu, Lei Pei, Xiaofeng Wang, Xiangtao Zheng, Song Yang, Shanzhi Zhao, Dan Xu, Ranran Li, Zhitao Yang, Enqiang Mao, Erzhen Chen, Ying Chen

**Affiliations:** ^1^ Department of Emergency, Ruijin Hospital, Shanghai Jiao Tong University School of Medicine, Shanghai, China; ^2^ Department of Critical Care Medicine, Ruijin Hospital, Shanghai Jiao Tong University School of Medicine, Shanghai, China

**Keywords:** sepsis, Mendelian randomization, inflammatory factors, metabolites, Axin1, IL-2, FGF-19

## Abstract

Sepsis, a life-threatening condition, involves complex interactions among metabolic alterations, inflammatory mediators, and host responses. This study utilized a bidirectional Mendelian randomization approach to investigate the causal relationships between 1400 metabolites and sepsis, and the mediating role of inflammatory factors. We identified 36 metabolites significantly associated with sepsis (p < 0.05), with AXIN1, FGF-19, FGF-23, IL-4, and OSM showing an inverse association, suggesting a protective role, while IL-2 exhibited a positive correlation, indicating a potential risk factor. Among these metabolites, Piperine and 9-Hydroxystearate demonstrated particularly interesting protective effects against sepsis. Piperine’s protective effect was mediated through its interaction with AXIN1, contributing to a 16.296% reduction in sepsis risk. This suggests a potential pathway where Piperine influences sepsis outcomes by modulating AXIN1 levels. 9-Hydroxystearate also exhibited a protective role against sepsis, mediated through its positive association with FGF-19 and negative association with IL-2, contributing 9.436% and 12.565%, respectively, to its protective effect. Experimental validation confirmed significantly elevated IL-2 levels and reduced FGF-19, AXIN1, piperine, and 9-hydroxyoctadecanoic acid levels in sepsis patients compared to healthy controls. Piperine levels positively correlated with AXIN1, while 9-hydroxyoctadecanoic acid levels negatively correlated with IL-2 and positively correlated with FGF-19, supporting the Mendelian randomization findings. Our findings provide insights into the molecular mechanisms of sepsis, highlighting the unique roles and contributions of specific metabolites and their interactions with inflammatory mediators. This study enhances our understanding of sepsis pathophysiology and opens avenues for targeted therapeutic interventions and biomarker development for sepsis management. However, further research is essential to validate these pathways across diverse populations and fully explore the roles of these metabolites in sepsis.

## 1 Introduction

Sepsis, defined as a life-threatening condition arising from dysregulated host responses to infection, presents a formidable challenge in healthcare, frequently necessitating admission to intensive care units (1). The 2017 Global Burden of Diseases, Injuries, and Risk Factors Study highlights the substantial, yet often overlooked, global impact of sepsis. As a leading cause of hospital mortality worldwide, it accounts for millions of new cases annually (2). Despite medical advancements, the mortality rate associated with sepsis remains alarmingly high, emphasizing the critical need for enhanced diagnostic and therapeutic approaches. The clinical management of sepsis is complicated by its heterogeneous nature, rapid progression, and the absence of specific early detection markers.

The pathophysiology of sepsis is highly complex, involving a dysregulated host response to infection that leads to life-threatening organ dysfunction (3). The complex interplay between the host’s immune system, inflammatory cascades, coagulation abnormalities, and microcirculatory dysfunction contributes to the diverse clinical manifestations of sepsis (4). These pathophysiological processes are closely intertwined with metabolic alterations and changes in inflammatory mediator levels, which play crucial roles in the development and progression of sepsis.

Metabolic alterations are a key feature of sepsis pathogenesis. The dysregulated host response to infection leads to significant changes in energy metabolism. Studies have identified significant changes in the levels of various metabolites in sepsis patients, such as increased lactate (5) and altered levels of amino acids and lipids (6). For instance, 3-hydroxybutyrate, a ketone body, has been shown to have anti-inflammatory and protective effects in sepsis. In a mouse model of lipopolysaccharide (LPS)-induced sepsis, oral administration of a ketone ester that increased 3-hydroxybutyrate levels significantly protected mice against systemic inflammation and organ dysfunction, including cardiac and renal dysfunction (7). Another example of a metabolite involved in sepsis pathogenesis is succinate, a tricarboxylic acid (TCA) cycle intermediate, which has been shown to accumulate in sepsis and contribute to the regulation of inflammatory responses (8). Succinate accumulation has been linked to the stabilization of hypoxia-inducible factor-1α (HIF-1α) and the production of pro-inflammatory cytokines, such as IL-1β (9). These metabolic disturbances not only reflect the host’s response to infection but also contribute to organ dysfunction and adverse outcomes in sepsis.

Inflammatory mediators play a crucial role in the development and progression of sepsis. Pro-inflammatory cytokines, such as IL-1β, IL-6, and TNF-α, are markedly elevated in sepsis and contribute to widespread inflammation, tissue damage, and organ failure (10, 11). For instance, IL-1β, produced by activated macrophages and monocytes, mediates sepsis-induced organ dysfunction, such as cardiomyopathy, and inhibition of the NLRP3/IL-1β axis has been shown to be protective in animal models (10). On the other hand, anti-inflammatory cytokines, like IL-4 and IL-10, attempt to counterbalance the excessive inflammatory response but may contribute to immunosuppression in sepsis (12, 13). IL-4 can downregulate pro-inflammatory cytokine production and promote alternative macrophage activation (12). IL-10, a potent anti-inflammatory cytokine, is secreted by macrophages during inflammation and counteracts the effects of pro-inflammatory mediators, such as TNF-α, leading to decreased oxidative stress (13).

The complex interplay between metabolic alterations and inflammatory mediators in sepsis remains to be fully elucidated. While some studies have identified associations between specific metabolites and inflammatory factors, such as the link between succinate and IL-1β production (9), the precise mechanisms and the collective impact of these interactions on sepsis outcomes are not well understood. A profound molecular understanding of sepsis is essential for improving its diagnosis, prognosis, and treatment. Elucidating the complex molecular pathways and identifying key biomarkers could transform the management of sepsis, leading to personalized therapeutic interventions (14). Investigating the interplay between metabolites and inflammatory factors in sepsis may provide valuable insights into its pathophysiology and help identify novel therapeutic targets.

Mendelian randomization (MR) studies, gaining traction alongside the evolution of genome-wide association studies, offer a novel approach to discern causal relationships (15). Increasingly, MR studies are shedding light on the exposure factors tied to the pathogenesis and prognosis of sepsis (16–18). MR stands out as a pivotal methodology, enabling the dissection of the intricate relationship between inflammatory mediators and metabolites in sepsis. This method holds promise in revolutionizing our understanding of sepsis and guiding the development of personalized treatment strategies. This study aims to elucidate the mediator role of inflammatory factors in the interaction between metabolites and sepsis, employing a bidirectional MR approach to provide potential insights into sepsis pathophysiology and therapeutic targets.

## 2 Materials and methods

### 2.1 Study design and data sources

**Figure 1:**
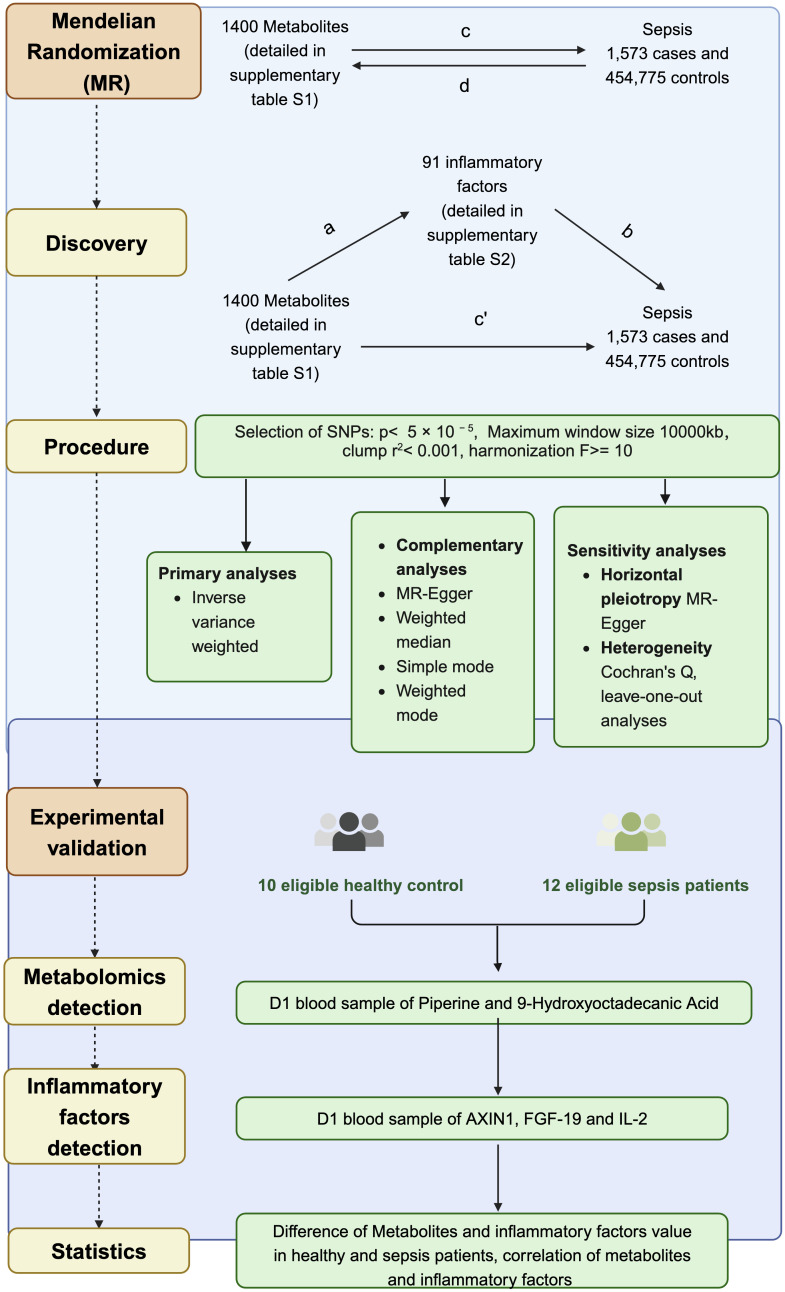
Schematic representation of the study design and analysis workflow. Bidirectional MR was performed to investigate the causal relationships between 1400 metabolites, 91 inflammatory factors, and sepsis using genetic data from 1,573 sepsis cases and 454,775 controls. The overall impact of metabolites on sepsis (c) was decomposed into the direct impact (c′) and the indirect impact mediated by inflammatory factors (a × b).

This study made use of extensive GWAS summary datasets, with the informed consent of participants obtained during the original studies. Our reliance on summary-level statistics negated the need for additional ethical approval. We utilized a bidirectional two-sample MR approach to investigate the mutually causal relationship between metabolites and sepsis, with a particular emphasis on understanding the mediating role of inflammatory factors. **Figure 1** illustrates the procedure using a flowchart. This observational study adhered to the Strengthening the Reporting of Observational Studies in Epidemiology using Mendelian Randomization (STROBE-MR) guidelines, with the checklist provided in the **Supplementary Table S1**. The utilized data, publicly accessible and predominantly of European ancestry, included genetic associations for sepsis sourced from the IEU Open GWAS project, encompassing 1,573 cases and 454,775 controls (19). GWAS data for 1400 metabolites factors can also be accessed through the IEU Open GWAS project (https://gwas.mrcieu.ac.uk/) with GWAS IDs (**Supplementary Table S2**) (20). The genetic associations of 91 inflammatory factors were derived from in the IEU Open GWAS project with GWAS IDs (**Supplementary Table S3**) (21).

### 2.2 Instrumental variable selection and data preparation

The analysis incorporated multiple exposure factors, identified via their respective GWAS IDs. We retrieved corresponding genetic instruments single nucleotide polymorphisms (SNPs) along with their associations for both the exposure and outcome. SNP data included beta coefficients, standard errors, allele details, frequencies, p-values, and sample sizes. Similarly, outcome data comprised the corresponding SNP associations. Rigorous criteria were employed to select instrumental variables (IVs) fulfilling three key assumptions. Given the limited number of available IVs, we adjusted our SNP selection threshold to p < 5 × 10^-5^ to capture a broader range of potentially relevant genetic instruments. Linkage disequilibrium clustering was executed using a window of 10,000 kb and an r^2^ threshold of < 0.001, based on the 1000 Genomes Project’s European samples. SNPs that were palindromic or ambiguous were excluded from the analysis. Data harmonization was meticulously conducted to ensure uniformity in the effect direction and allele coding across all SNPs. The instrumental strength of each SNP was rigorously assessed using R² and F-statistics. We excluded SNPs with an F-statistic lower than 10 to maintain the robustness of our instrumental variables (**Supplementary Table S4**).

### 2.3 MR estimation

MR estimates were then computed, extracting Odds Ratios (ORs) and p-values for each exposure factor to pinpoint statistically significant associations (p < 0.05). Our analysis integrated a suite of methods to estimate causal effects accurately. The Inverse Variance Weighted (IVW) method utilizes a meta-analytical approach, aggregating the Wald ratios from each SNP to determine the combined causal effects. This method operates under the assumption that all SNPs are valid instrumental variables, allowing for precise and accurate estimations. To complement and validate these results, MR-Egger and the Weighted Median approach were also utilized. Each method is tailored to specific assumptions about the validity of instrumental variables.

### 2.4 Sensitivity analyses

Sensitivity analyses were conducted to validate the robustness of our findings. MR Steiger filtering determined the causal direction for each SNP relative to the exposure and outcome. SNP homogeneity was assessed using Cochran’s Q statistic and funnel plots, while horizontal pleiotropy was examined via MR-Egger intercept and MR-PRESSO methods, with outliers removed for re-evaluation. Persistent heterogeneity was addressed using a random effects model. Additionally, a leave-one-out analysis was performed to assess the impact of individual SNPs.

### 2.5 Mediation analysis of intermediate effect

A two-sample MR assessed mutual causality between metabolites and sepsis, followed by inflammatory factors and sepsis. After deriving MR estimates, statistically significant associations were identified (p < 0.05) using the instrumental variable method. The relationship between statistically significant metabolites and inflammatory factors was done. The total effect, representing bidirectional MR between inflammatory factors and sepsis, was initially designated. A two-step bidirectional MR design then facilitated mediation analysis to examine if metabolites mediate the pathway from inflammatory factors to sepsis. The total effect was broken down into mediating effects and indirect effects mediated through inflammatory factors. To determine the mediation percentage, divide the indirect effect by the total effect.

### 2.6 Patients enrolled

Patients diagnosed with sepsis in the emergency department of Shanghai Ruijin Hospital from October 31, 2021, to May 20, 2022 were enrolled in this study. Inclusion criteria were: (1) age 18–90 years, (2) meeting the diagnostic criteria for sepsis 3.0, and (3) hospital stay exceeding 24 hours. Exclusion criteria consisted of: (1) discharge or death within 24 hours of admission, (2) participation in other clinical trials, (3) requirement for emergency surgery post-admission, (4) presence of malignant tumor, (5) pregnancy or lactation.

### 2.7 Metabolomics analysis

Untargeted metabolomics analysis was performed on plasma samples to detect Piperine. The samples stored at -80°C were thawed on ice and vortexed for 10 s. 50 μL of sample and 300 μL of extraction solution (ACN: Methanol = 1:4, V/V) containing internal standards were added into a 2 mL microcentrifuge tube. The sample was vortexed for 3 min and then centrifuged at 12000 rpm for 10 min (4°C). 200 μL of the supernatant was collected and placed in -20°C for 30 min, and then centrifuged at 12000 rpm for 3 min (4°C). A 180 μL aliquot of supernatant was transferred for LC-MS analysis. The LC-MS system was operated under the following conditions: UPLC column, Waters ACQUITY UPLC HSS T3 C18 (1.8 µm, 2.1 mm*100 mm); column temperature, 40°C; flow rate, 0.4 mL/min; injection volume, 2 μL; solvent system, water (0.1% formic acid): acetonitrile (0.1% formic acid). The column was eluted with 5% mobile phase B (0.1% formic acid in acetonitrile) at 0 minute followed by a linear gradient to 90% mobile phase B over 11 minutes, held for 1 minute, and then returned to 5% mobile phase B within 0.1 minute, held for 1.9 minutes.

Due to the absence of 9-Hydroxystearate in the untargeted metabolomics analysis, we opted for targeted metabolomics to quantify its upstream metabolite, 9-Hydroxyoctadecanic Acid, as a surrogate. A standard solution of 9-Hydroxyoctadecanic Acid was prepared and serially diluted to generate a calibration curve. For targeted metabolomics analysis, 100 μL of each plasma sample was mixed with 1 mL of methanol, vortexed, and centrifuged to collect the supernatant. The solid-phase extraction column was activated using 1 mL of methanol and 1 mL of 0.1% formic acid solution, followed by the addition of 4 mL of 0.1% formic acid solution to the supernatant. The sample was then loaded onto the column and washed sequentially with 1 mL of formic acid solution and 1 mL of 15% ethanol solution. This process was repeated once more. Chromatographic separation was performed on a BEH C18 column (2.1 mm × 100 mm × 1.7 μm) with a column temperature of 40°C and an injection volume of 10 μL. The mobile phases consisted of water (A) and methanol (B), and a gradient elution program was employed. Mass spectrometry analysis was conducted using electrospray ionization (ESI) in negative mode, with a drying gas temperature of 350°C, a drying gas flow rate of 10 L/min, and a capillary voltage of 4000 V.

### 2.8 Enzyme-Linked Immunosorbent Assay (ELISA) for inflammatory factors

The levels of inflammatory factors AXIN1, FGF-19, and IL-2 in plasma samples were determined using commercial ELISA kits (Mlbio, ml564859V, ml038426V, ml058063V) following the manufacturer’s instructions. Briefly, 96-well microplates were coated with capture antibodies specific for each target protein and incubated overnight at 4°C. After washing with PBS containing 0.05% Tween-20 (PBST), the plates were blocked with 1% BSA in PBS for 1 hour at room temperature. Standards and plasma samples (100 μL) were added to the wells and incubated for 2 hours at room temperature. The plates were then washed with PBST, and biotinylated detection antibodies were added, followed by incubation for 1 hour at room temperature. After another washing step, streptavidin-horseradish peroxidase (HRP) conjugate was added, and the plates were incubated for 30 minutes at room temperature. The plates were washed again, and 3,3’,5,5’-tetramethylbenzidine (TMB) substrate solution was added to each well. The reaction was stopped after 15 minutes by adding 2 M sulfuric acid, and the optical density was measured at 450 nm using a microplate reader. The concentrations of AXIN1, FGF-19, and IL-2 in the plasma samples were calculated based on the standard curves generated using recombinant proteins provided in the ELISA kits. All samples were analyzed in duplicate, and the mean values were used for statistical analysis.

### 2.9 Statistical analysis

Statistical analyses were performed using R software (Version 4.1.3). Normality of the data was assessed using the Shapiro-Wilk test. Comparisons of plasma metabolite and inflammatory factor levels between healthy controls and sepsis patients were conducted using the independent samples t-test for normally distributed data or the Mann-Whitney U test for non-normally distributed data. The linear relationships between plasma metabolites and inflammatory factors were assessed using Pearson’s correlation coefficient for normally distributed data or Spearman’s rank correlation coefficient for non-normally distributed data. Correlation matrices were generated using the “corrplot” package in R to visualize the relationships between variables, and the significance of the correlations was determined based on two-tailed p-values. Data visualization was performed using the “ggplot2” package in R. A p-value < 0.05 was considered statistically significant for all analyses.

## 3 Results

### 3.1 Association of metabolites with sepsis

**Figure 2:**
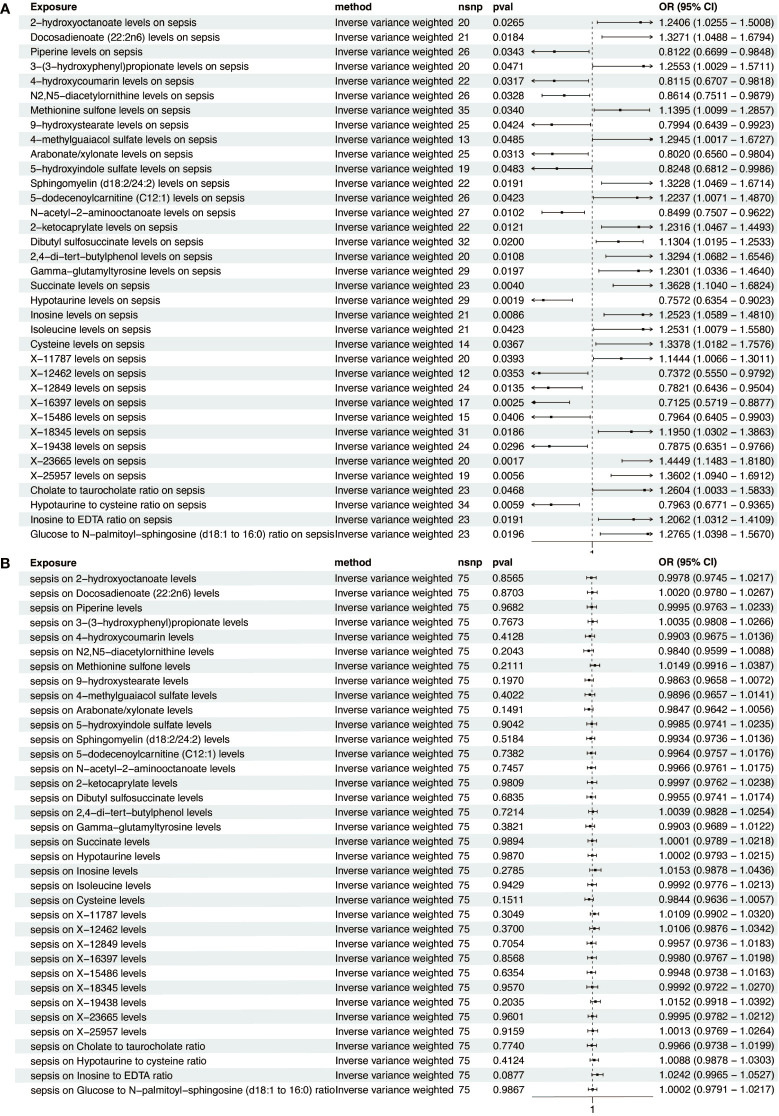
Forest plot of metabolite-sepsis causal associations. **(A)** This plot displays the significant causal links of various metabolites with sepsis risk. **(B)** This plot demonstrates the significant causal influences of sepsis on different metabolites.

To investigate the influence of 1400 metabolites on sepsis, we primarily used the IVW method for analysis. The 36 significant metabolites correlated with sepsis are listed in **Figure 2A**. To ascertain the causal direction, reverse MR was performed. Sepsis, as an exposure, showed no effect on metabolites (**Figure 2B**). Cochran’s Q-test revealed no significant heterogeneity (**Supplementary Table S5**). MR-Egger intercept test showed no pleiotropy (**Supplementary Table S6**).

### 3.2 Association of inflammatory factors with sepsis

**Figure 3:**
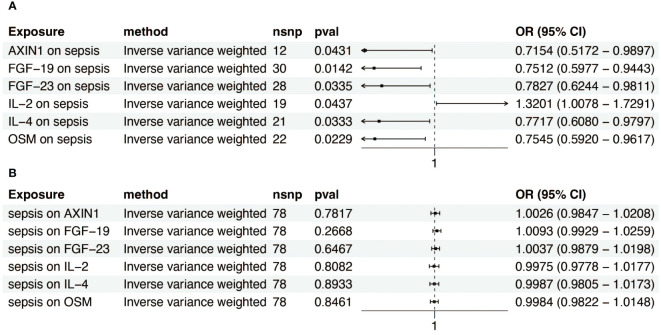
Forest plot of inflammatory factor-sepsis causal associations. **(A)** This plot depicts the significant causal connections between a range of inflammatory factors and sepsis risk. **(B)** This plot shows the significant causal impacts of sepsis on various inflammatory factors.

To investigate the influence of 91 inflammatory factors on sepsis, we primarily used the IVW method for analysis. The IVW analysis revealed a notable inverse association between FGF-19 and sepsis (OR = 0.751, 95% CI (confidence interval) = 0.598–0.944). Similar negative associations were observed for AXIN1 (OR = 0.715, CI = 0.517–0.990), FGF-23 (OR = 0.783, CI = 0.624–0.981), IL-4 (OR = 0.772, CI = 0.608–0.980), and OSM (OR = 0.755, CI = 0.592–0.962), whereas IL-2 showed a positive correlation with sepsis (OR = 1.320, CI = 1.008–1.729) (**Figure 3A**). Results for other inflammatory factors that did not reach statistical significance are presented in **Supplementary Table S7**. To ascertain the causal direction, reverse MR was performed. Sepsis, as an exposure, showed no effect on inflammatory factors (**Figure 3B**). Cochran’s Q-test, revealed no significant heterogeneity (**Supplementary Table S5**). MR-Egger intercept test showed no pleiotropy (**Supplementary Table S6**).

### 3.3 Association of sepsis-relevant metabolites with inflammatory factors

**Figure 4:**
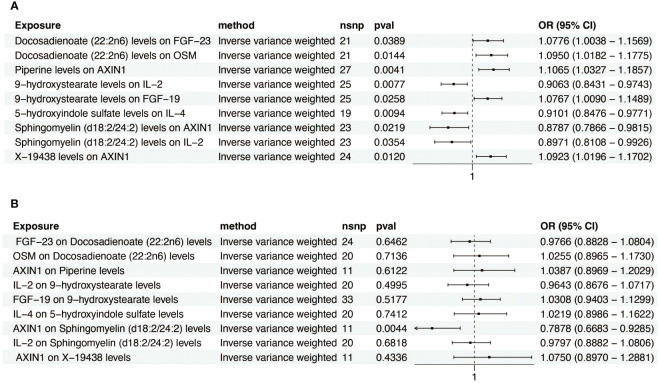
Forest plot of metabolite-inflammatory factor causal relationships. **(A)** This plot presents the significant causal interactions of key metabolites with inflammatory factors. **(B)** This plot reveals the significant causal influences of prominent inflammatory factors on metabolites.

We next investigated the influence of 36 metabolites on FGF-19, FGF-23, IL-4, IL-2, OSM, and AXIN1. IVW analysis revealed significant correlations between these inflammatory factors and various metabolites (**Figure 4A**). To ascertain the causal direction, reverse MR was performed, AXIN1 as exposure, was causally associated with Sphingomyelin (d18:2/24:2) levels, with other inflammatory factors, as exposure, showed no effect on metabolites (**Figure 4B**). Cochran’s Q-test, revealed no significant heterogeneity in the causal relationship (**Supplementary Table S4**). MR-Egger intercept test showed no pleiotropy (**Supplementary Table S5**).

### 3.4 Percentage of the link between metabolites and sepsis accounted for by inflammatory factors

**Figure 5:**
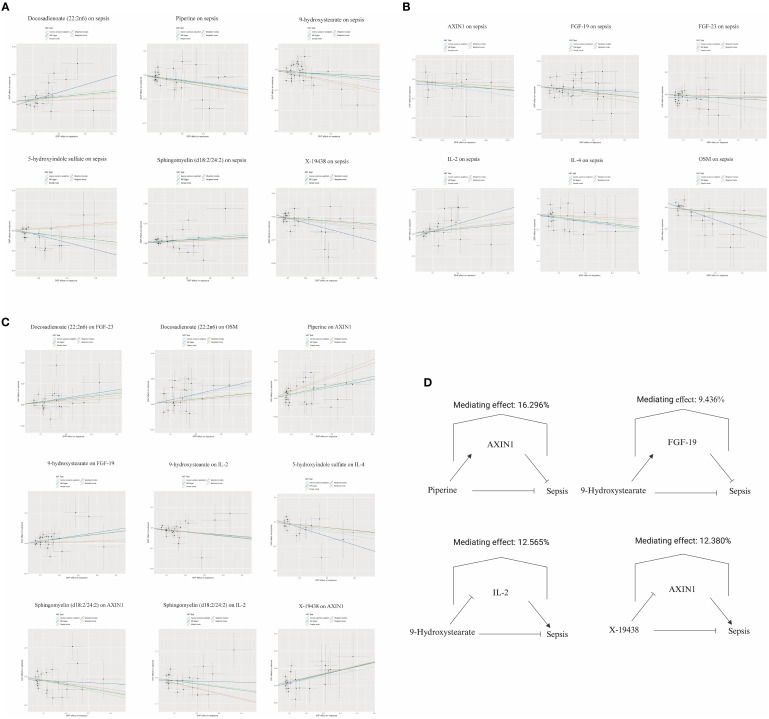
Diagram of mediating effects in metabolite-inflammatory factor-sepsis Interactions. **(A)** Scatter plot illustrating the causal effects of specific metabolites on sepsis. **(B)** Scatter plot demonstrating the causal effects of selected inflammatory factors on sepsis. **(C)** Scatter plot showing the causal effects of particular metabolites on inflammatory factors. **(D)** This diagram provides a visual summary of the mediation analysis, highlighting how specific inflammatory factors influence the relationship between metabolites and sepsis.

Our analysis identified that Docosadienoate (22:2n6) and Sphingomyelin (d18:2/24:2) were positively correlated with sepsis. Conversely, Piperine, 9-hydroxystearate, 5-hydroxyindole sulfate, and X-19438 showed negative correlations with sepsis (**Figure 5A**). Inflammatory factors such as AXIN-1, FGF-19, FGF-23, IL-4, and OSM were negatively causally associated with sepsis, while IL-2 was positively associated (**Figure 5B**). We observed that metabolites were causally associated with inflammatory factors (**Figure 5C**). Leave-one-out analysis indicated no significant bias introduced by any single SNP (**Supplementary Figure S1**). Assessing inflammatory factors as mediators between metabolites and sepsis, we found that while Docosadienoate (22:2n6) was correlated with sepsis, it was also associated with FGF-23 and OSM. However, since FGF-23 and OSM were negatively associated with sepsis, they are unlikely to mediate the increased sepsis risk associated with Docosadienoate (22:2n6). The association of Piperine with increased AXIN1 correlated with a reduced sepsis risk, contributing 16.296% to Piperine’s protective effect. Similarly, 9-hydroxystearate’s association with increased FGF-19 and decreased IL-2 correlated with a reduced sepsis risk, contributing 9.436% and 12.565% respectively to the protective effect of 9-hydroxystearate. The negative association of 5-hydroxyindole sulfate with sepsis, and its association with increased IL-4 (which is negatively associated with sepsis), suggests that IL-4 is not a mediator for 5-hydroxyindole sulfate’s protective effect against sepsis. Sphingomyelin (d18:2/24:2) was positively associated with sepsis and negatively associated with IL-2, but since IL-2 was positively associated with sepsis, it is unlikely to be a mediator. The association of X-19438 with increased AXIN1 correlated with a reduced sepsis risk, contributing 12.380% to the protective effect of X-19438 (**Figure 5D**).

### 3.5 Experimental validation of the associations between metabolites, inflammatory factors, and sepsis

**Figure 6:**
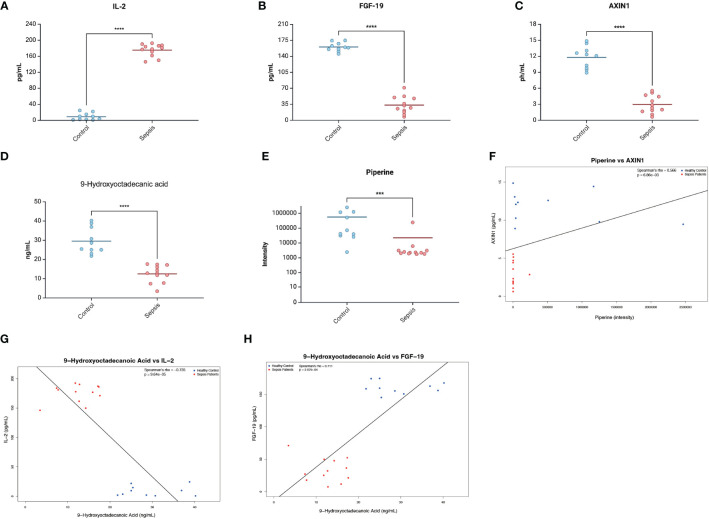
Experimental validation of associations between metabolites, inflammatory factors, and sepsis. **(A–C)** Plasma levels of inflammatory factors IL-2 **(A)**, FGF-19 **(B)**, and AXIN1 **(C)** in healthy controls and sepsis patients. **(D, E)** Plasma levels of metabolites 9-hydroxyoctadecanoic acid **(D)** and piperine **(E)** in healthy controls and sepsis patients. Data are presented as mean ± SD. ***p < 0.001, ****p < 0.0001. **(F–H)** Correlation analysis between metabolites and inflammatory factors in healthy controls (blue dots) and sepsis patients (red dots). **(F)** Correlation between piperine and AXIN1 expression (Spearman's rho = 0.566, p = 6.86e-03). **(G)** Correlation between 9-hydroxyoctadecanoic acid and IL-2 levels (Spearman's rho = -0.735, p = 9.64e-05). **(H)** Correlation between 9-hydroxyoctadecanoic acid and FGF-19 levels (Spearman's rho = 0.711, p = 2.07e-04).

To validate the findings from the bidirectional Mendelian randomization analysis, we compared the levels of key metabolites (piperine and 9-hydroxyoctadecanoic acid) and inflammatory factors (IL-2, FGF-19, and AXIN1) in plasma samples from sepsis patients on the first day of hospital admission and healthy controls. The results showed that sepsis patients exhibited significantly elevated levels of the inflammatory factor IL-2 and significantly reduced levels of FGF-19 and AXIN1 compared to healthy controls (**Figures 6A–C**). Furthermore, sepsis patients had significantly lower levels of piperine and 9-hydroxyoctadecanoic acid compared to healthy controls (**Figures 6D, E**).

Linear correlation analysis revealed significant associations between the levels of metabolites and inflammatory factors in the combined population of sepsis patients and healthy controls. Specifically, piperine levels were positively correlated with AXIN1 (rho=0.566, p=6.86*10^-3^) (**Figure 6F**). Additionally, 9-hydroxyoctadecanoic acid levels were negatively correlated with IL-2 (rho=-0.735, p= 9.64*10^-5^) (**Figure 6G**). Meanwhile, 9-hydroxyoctadecanoic acid was positively correlated with FGF-19 (rho=0.711, p=9.64*10^-5^) (**Figure 6H**).

These experimental findings support the results of the bidirectional Mendelian randomization analysis, providing further evidence for the potential causal relationships between the metabolites piperine and 9-hydroxyoctadecanoic acid, the inflammatory factors IL-2, FGF-19, and AXIN1, and the risk of sepsis. The observed associations suggest that these metabolites and inflammatory factors may play important roles in the pathophysiology of sepsis and could serve as potential therapeutic targets or biomarkers for sepsis management.

## 4 Discussion

Our study delves into the complex interplay between inflammatory mediators, metabolic changes, and their collective impact on sepsis pathogenesis. The roles of inflammatory factors and metabolites are pivotal in sepsis, yet their distinct pathways necessitate a thorough examination of both individual and combined effects. Inflammatory mediators, such as cytokines, proteases, and lipid mediators, play a crucial role in exacerbating tissue damage and enhancing host susceptibility to infection (22). They trigger various signaling pathways that can lead to tissue damage and potentially multi-organ failure. Concurrently, sepsis-induced metabolic alterations, involving shifts in macronutrient metabolism, critically alter the patient’s metabolic state (23). These alterations affect energy production and utilization, thereby influencing survival and recovery outcomes. Metabolic alterations can significantly impact inflammatory responses. For example, melatonin and its metabolites exhibit potent antioxidant and anti-inflammatory properties that can mitigate inflammation-induced mitochondrial dysfunction and oxidative stress (24). Conversely, inflammatory factors are known to modulate metabolic pathways, as evidenced by changes in the expression and activity of drug-metabolizing enzymes (25). However, the intricate process through which metabolites influences sepsis via inflammatory factors alteration remains poorly understood.

In our MR analysis, we discovered that piperine influences sepsis progression by affecting AXIN1. Our findings suggest that elevated levels of piperine are associated with a reduced risk of sepsis. Previous studies have highlighted piperine’s potential for neuroprotection in sepsis-associated encephalopathy, as demonstrated in animal models subjected to Cecal Ligation and Puncture (26). Additionally, several studies have reported that piperine inhibits inflammatory factors in various diseases. For instance, it can inhibit pyroptosis and the release of interleukin-1β in response to ATP stimulation and bacterial infection (27), and it also reduces Lithocholic Acid-induced Interleukin-8 production in human colorectal cancer cells by inhibiting Src/EGFR and reactive oxygen species (28). However, to date, no studies have reported on the impact of piperine on AXIN-1. Our study indicates that the association of piperine with reduced sepsis risk is partly due to AXIN-1, accounting for approximately 16.296% of this protective effect. AXIN-1, a component of the β-catenin degradation complex, regulates the Wnt signaling pathway, which recent studies have linked to sepsis (29). Wnt signaling inhibitors, such as Wnt-C59 and LGK974, have been shown to modulate inflammatory responses in sepsis, thereby improving prognosis (30, 31). APC proteins, known for their role in negatively regulating the Wnt pathway by facilitating β-catenin degradation, are crucial in this context. Mutations in APC result in the stabilization of β-catenin and activation of the Wnt pathway. Axin1, similar to APC, promotes β-catenin degradation. The detailed mechanism by which piperine reduces sepsis risk via AXIN1 warrants further investigation.

9-Hydroxystearate, a salt form of 9-hydroxyoctadecanoic acid, is an endogenous cellular lipid. Several studies have demonstrated its inhibitory effect on cancers, such as colon cancer (32) and Osteosarcoma (33). Recent research has also shown that 9-Hydroxystearic acid can influence the inflammatory process. Research has shown that it possesses anti-inflammatory effects, notably suppressing cytokines like IL-1β and IL-6 triggered by LPS in RAW 264.7 cells (34). However, the relationship between 9-Hydroxystearic acid and FGF-19, as well as IL-2, is currently underreported. In our study, we found that the association of 9-hydroxystearate levels with increased FGF-19 correlated with a reduced sepsis risk, contributing 9.436% to the protective effect of 9-hydroxystearate. Similarly, its association with decreased IL-2 levels correlated with a reduced sepsis risk, contributing 12.565% to its protective effect. FGF-19, primarily synthesized in the ileum following Farnesoid X Receptor activation, plays a role in reducing hepatic bile acid production (35). FGF-19 has been shown to exhibit anti-inflammatory effects, with studies indicating lower levels of FGF-19 in Inflammatory Bowel Disease (IBD), resulting from intestinal inflammation, compromised barrier function, and impaired bile acid absorption (36). IL-2 acts as a pro-inflammatory cytokine, contributing to the pathogenesis of sepsis by participating in the systemic inflammatory response. IL-2 induces IL-17 production by lung granular γδ T cells, leading to increased IL-17 synthesis and neutrophil recruitment (37). The detailed mechanism by which 9-Hydroxystearate reduces sepsis risk via FGF-19 and IL-2 requires further investigation. In our study, X-19438 was found to inhibit the occurrence of sepsis through a positive correlation with AXIN1. However, the exact nature and physiological role of the metabolite X-19438 remain to be clearly defined. Future research is needed to elucidate the physiological functions of X-19438.

In our study, although some metabolites demonstrated causal associations with inflammatory factors, the direction of causality and the consistency of these inflammatory factors with sepsis were not aligned, indicating that these inflammatory factors did not serve as mediators. Docosadienoate (22:2n6), a polyunsaturated fatty acid from the Omega-6 family, exhibited a positive causal association with sepsis in our analysis. Previous research has shown that Omega-6 polyunsaturated fatty acids can have pro-inflammatory effects, in contrast to the anti-inflammatory actions of oleic acid and Omega-3 polyunsaturated fatty acids (38). The opposing causal associations of Docosadienoate (22:2n6) with FGF-23 and OSM suggest that it does not act as a mediator. The increased sepsis risk associated with Docosadienoate (22:2n6) may rely on other factors, necessitating further investigation. 5-Hydroxyindole sulfate, produced through the same metabolic pathway as serotonin, is a downstream product of serotonin metabolism. Serotonin is converted into 5-Hydroxyindoleacetic acid, which is then further transformed into 5-Hydroxyindole sulfate. Sulfation, a common phase II metabolic process, typically increases the water solubility of compounds, thereby facilitating excretion. The positive correlation of 5-Hydroxyindole sulfate with the risk of sepsis may be attributed to the properties of 5-Hydroxyindoleacetic acid. Studies have indicated that 5-Hydroxyindoleacetic acid levels are significantly higher in the plasma of patients with septic shock compared to those with sepsis alone. As a metabolite of a neurotransmitter, it can increase vascular permeability, leading to shock (39). In our study, the opposing causal relationship of 5-Hydroxyindole sulfate with IL-4 suggests that it does not act as a mediator, and its role in reducing sepsis risk may depend on other factors. Sphingomyelin, a crucial component of vascular endothelium, is implicated in the early pathogenesis of sepsis. The attack on endothelial cells by inflammatory factors leads to the release of endothelium-associated sphingomyelins, which explains why elevated plasma levels are a risk factor for sepsis. While direct studies on Sphingomyelin (d18:2/24:1) are limited, research indicates elevated levels of this metabolite in the cerebrospinal fluid of Parkinson’s patients, potentially reflecting pathological damage due to blood-brain barrier leakage (40). In our study, no inflammatory mediators were identified as intermediaries for Sphingomyelin (d18:2/24:2) in promoting sepsis, suggesting the involvement of other intermediary factors.

The experimental validation of the bidirectional Mendelian randomization findings provides crucial insights into the complex interplay between metabolites, inflammatory factors, and sepsis. By demonstrating significant alterations in the levels of key metabolites (piperine and 9-hydroxyoctadecanoic acid) and inflammatory factors (IL-2, FGF-19, and AXIN1) in sepsis patients compared to healthy controls, this study highlights the potential role of these molecules in the pathophysiology of sepsis. The significant correlations observed between piperine and AXIN1, as well as 9-hydroxyoctadecanoic acid and IL-2 and FGF-19, further support the intricate relationships between metabolites and inflammatory factors in the context of sepsis. These findings suggest that the protective effects of piperine and 9-hydroxyoctadecanoic acid against sepsis may be mediated through their influence on the levels of AXIN, IL-2 and FGF-19, respectively.

In our study, we identified several inflammatory factors, including FGF-19, AXIN1, FGF-23, IL-4, OSM, and IL-2, that showed statistically significant associations with sepsis risk using the IVW method in MR analyses. However, it is notable that some well-established inflammatory mediators in sepsis, such as TNF-α, IL-1β and IL-17, did not show significant associations with sepsis risk in our analyses. The lack of significant associations between these inflammatory mediators and sepsis risk in our study could be attributed to several factors. First, our study may have been underpowered to detect causal associations for these specific inflammatory mediators, particularly if the effect sizes of these associations were small. Second, the inflammatory response in sepsis is a complex process involving the interaction of multiple inflammatory mediators and signaling pathways, and the effects of individual inflammatory mediators may be masked by other factors or may exhibit non-linear relationships with sepsis risk, which are not well captured by MR analyses that primarily assess linear relationships. Furthermore, the role of these inflammatory mediators in sepsis may be context-dependent or may vary across different stages of the disease. For example, TNF-α and IL-1β are typically associated with the early, hyperinflammatory phase of sepsis (41), while IL-17 may be more involved in the later, immunosuppressive phase (42). Therefore, the impact of these inflammatory mediators on sepsis risk may not be adequately captured by the genetic variants used as instrumental variables in our MR analyses.

Our study, exploring the interplay between metabolites, inflammatory factors, and sepsis, has several limitations. The MR approach, while effective for establishing causal relationships, depends on the validity of IVs. Despite stringent selection, potential residual confounding due to linkage disequilibrium or pleiotropy, especially regarding the metabolites and inflammatory factors studied, may affect our findings’ accuracy. Another limitation is related to the selection of SNPs based on the p-value threshold. In our analysis, we adjusted the SNP selection threshold to p < 5 × 10^-5^ to obtain a broader range of genetic instruments, as the more stringent genome-wide significance threshold of p < 5 × 10^-8^ would have resulted in a limited number of SNPs for some exposures (e.g., only one SNP for 9-Hydroxystearate and X-19438, and one SNP for AXIN-1). While relaxing the p-value threshold allowed us to include more SNPs and increase the statistical power of our analysis, it may also have introduced potential weak instrument bias (43). Weak instruments can bias the causal estimate toward the observational association, leading to an increased risk of false-positive findings (44). Additionally, our analysis primarily uses data from European ancestry individuals, limiting the generalizability of our results to other ethnic groups with different metabolic and inflammatory responses. Furthermore, focusing on specific metabolites and inflammatory factors does not encompass the entire spectrum of molecules involved in sepsis pathophysiology. Our findings, therefore, represent a segment of the complex sepsis landscape. Also, reliance on public GWAS databases might introduce biases affecting data quality and reporting, impacting result reliability. Finally, translating our findings into clinical practice requires clinical validation through experimental studies and trials. A comprehensive understanding of the underlying molecular mechanisms and their clinical application is crucial for developing effective sepsis treatments.

## 5 Conclusion

Our study has identified significant interactions between specific metabolites and inflammatory factors in the context of sepsis. Piperine, in particular, demonstrated a protective effect against sepsis, mediated through its interaction with AXIN1, contributing to a 16.296% reduction in sepsis risk. This finding suggests a potential pathway where Piperine influences sepsis outcomes by modulating AXIN1 levels. Additionally, 9-Hydroxystearate exhibited a dual protective role against sepsis, positively associated with FGF-19 and negatively with IL-2, contributing 9.436% and 12.565% respectively to its protective effect. These results highlight the complex role of 9-Hydroxystearate in sepsis pathophysiology. The third metabolite, X-19438, while identified as significant, necessitates further research to elucidate its specific role and interactions with inflammatory factors in sepsis. Our findings provide valuable insights into the molecular mechanisms of sepsis and underscore the potential of targeted metabolic interventions in its management.

## Data availability statement

Publicly available datasets were analyzed in this study. This data can be found here: https://gwas.mrcieu.ac.uk/.

## Ethics statement

The research protocol for this retrospective study adhered to the principles outlined in the Declaration of Helsinki. This study was approved by the Ethics Committee of Ruijin Hospital (No.20210101) (**Supplementary Table S8**). The participants provided their written informed consent to participate in this study.

## Author contributions

FG: Data curation, Methodology, Writing – original draft. WL: Data curation, Methodology, Writing – original draft, Validation. LP: Data curation, Methodology, Writing – original draft. XW: Investigation, Writing – review & editing. XZ: Investigation, Writing – review & editing. SY: Investigation, Writing – review & editing. SZ: Investigation, Writing – review & editing. DX: Investigation, Writing – review & editing. RL: Investigation, Writing – review & editing. ZY: Writing – review & editing, Supervision. EM: Supervision, Writing – review & editing. EC: Writing – review & editing, Conceptualization, Funding acquisition. YC: Conceptualization, Writing – review & editing, Project administration.

## Supplementary material

Supplementary Figure 1 Leave-one-out stability analysis.

Supplementary Table 1 STROBE-MR.

Supplementary Table 2 Comprehensive list of 1400 metabolites.

Supplementary Table 3 Comprehensive list of 91 inflammatory factors.

Supplementary Table 4 Instrumental variables for exposure post-data harmonization.

Supplementary Table 5 Assessment of heterogeneity among SNPs.

Supplementary Table 6 Evaluation of pleiotropy among SNPs.

Supplementary Table 7 Mendelian randomization analysis results for inflammatory factors not significantly associated with sepsis risk.

Supplementary Table 8 Ethics approval document.

## References

[1] Evans L Rhodes A Alhazzani W Antonelli M Coopersmith CM French C. Surviving sepsis campaign: international guidelines for management of sepsis and septic shock 2021. Crit Care Med. (2021) 49:e1063–e143. doi: 10.1007/s00134-021-06506-y 34605781

[2] Gu X Zhou F Wang Y Fan G Cao B. Respiratory viral sepsis: epidemiology, pathophysiology, diagnosis and treatment. Eur Respir Rev. (2020) 29(157):200038. doi: 10.1183/16000617.0038-2020 32699026 PMC9489194

[3] Shankar-Hari M Phillips GS Levy ML Seymour CW Liu VX Deutschman CS. Developing a new definition and assessing new clinical criteria for septic shock: for the third international consensus definitions for sepsis and septic shock (Sepsis-3). JAMA. (2016) 315:775–87. doi: 10.1001/jama.2016.0289 PMC4910392 26903336

[4] Christaki E Giamarellos-Bourboulis EJ. The beginning of personalized medicine in sepsis: small steps to a bright future. Clin Genet. (2014) 86:56–61. doi: 10.1111/cge.12368 24579691

[5] Nedel WL Portela LV. Lactate levels in sepsis: don’t forget the mitochondria. Intensive Care Med. (2024) 50(7):1202–3. doi: 10.1007/s00134-024-07475-8 38780796

[6] Langley RJ Tsalik EL van Velkinburgh JC Glickman SW Rice BJ Wang C. An integrated clinico-metabolomic model improves prediction of death in sepsis. Sci Transl Med. (2013) 5:195ra95. doi: 10.1126/scitranslmed.3005893 PMC3924586 23884467

[7] Soni S Martens MD Takahara S Silver HL Maayah ZH Ussher JR. Exogenous ketone ester administration attenuates systemic inflammation and reduces organ damage in a lipopolysaccharide model of sepsis. Biochim Biophys Acta Mol Basis Dis. (2022) 1868:166507. doi: 10.1016/j.bbadis.2022.166507 35902007

[8] Liu H Zhang H Zhang X Chen Q Xia L. Role of succinic acid in the regulation of sepsis. Int Immunopharmacol. (2022) 110:109065. doi: 10.1016/j.intimp.2022.109065 35853278

[9] Mills EL Kelly B Logan A Costa ASH Varma M Bryant CE. Succinate dehydrogenase supports metabolic repurposing of mitochondria to drive inflammatory macrophages. Cell. (2016) 167:457–70.e13. doi: 10.1016/j.cell.2016.08.064 27667687 PMC5863951

[10] Busch K Kny M Huang N Klassert TE Stock M Hahn A. Inhibition of the NLRP3/IL-1beta axis protects against sepsis-induced cardiomyopathy. J Cachexia Sarcopenia Muscle. (2021) 12:1653–68. doi: 10.1002/jcsm.12763 PMC8718055 34472725

[11] Ling H Chen M Dai J Zhong H Chen R Shi F. Evaluation of qSOFA combined with inflammatory mediators for diagnosing sepsis and predicting mortality among emergency department. Clin Chim Acta. (2023) 544:117352. doi: 10.1016/j.cca.2023.117352 37076099

[12] Schrijver DP Roring RJ Deckers J de Dreu A Toner YC Prevot G. Resolving sepsis-induced immunoparalysis via trained immunity by targeting interleukin-4 to myeloid cells. Nat BioMed Eng. (2023) 7:1097–112. doi: 10.1038/s41551-023-01050-0 PMC10504080 37291433

[13] Sawoo R Dey R Ghosh R Bishayi B. Exogenous IL-10 posttreatment along with TLR4 and TNFR1 blockade improves tissue antioxidant status by modulating sepsis-induced macrophage polarization. J Appl Toxicol. (2023) 43:1549–72. doi: 10.1002/jat.4496 37177863

[14] Lodge S Litton E Gray N Ryan M Millet O Fear M. Stratification of sepsis patients on admission into the intensive care unit according to differential plasma metabolic phenotypes. J Proteome Res. (2024) 23:1328–40. doi: 10.1021/acs.jproteome.3c00803 PMC11002934 38513133

[15] Bowden J Holmes MV. Meta-analysis and Mendelian randomization: A review. Res Synth Methods. (2019) 10:486–96. doi: 10.1002/jrsm.1346 PMC6973275 30861319

[16] Allman PH Aban IB Tiwari HK Cutter GR. An introduction to Mendelian randomization with applications in neurology. Mult Scler Relat Disord. (2018) 24:72–8. doi: 10.1016/j.msard.2018.06.017 29960142

[17] Flatby HM Ravi A Damas JK Solligard E Rogne T. Circulating levels of micronutrients and risk of infections: a Mendelian randomization study. BMC Med. (2023) 21:84. doi: 10.1186/s12916-023-02780-3 36882828 PMC9993583

[18] Ponsford MJ Gkatzionis A Walker VM Grant AJ Wootton RE Moore LSP. Cardiometabolic traits, sepsis, and severe COVID-19: A mendelian randomization investigation. Circulation. (2020) 142:1791–3. doi: 10.1161/CIRCULATIONAHA.120.050753 PMC7594537 32966752

[19] Jiang L Zheng Z Fang H Yang J. A generalized linear mixed model association tool for biobank-scale data. Nat Genet. (2021) 53:1616–21. doi: 10.1038/s41588-021-00954-4 34737426

[20] Chen Y Lu T Pettersson-Kymmer U Stewart ID Butler-Laporte G Nakanishi T. Genomic atlas of the plasma metabolome prioritizes metabolites implicated in human diseases. Nat Genet. (2023) 55:44–53. doi: 10.1038/s41588-022-01270-1 36635386 PMC7614162

[21] Zhao JH Stacey D Eriksson N Macdonald-Dunlop E Hedman AK Kalnapenkis A. Genetics of circulating inflammatory proteins identifies drivers of immune-mediated disease risk and therapeutic targets. Nat Immunol. (2023) 24:1540–51. doi: 10.1038/s41590-023-01588-w PMC10457199 37563310

[22] Aziz M Jacob A Yang WL Matsuda A Wang P. Current trends in inflammatory and immunomodulatory mediators in sepsis. J Leukoc Biol. (2013) 93:329–42. doi: 10.1189/jlb.0912437 PMC3579020 23136259

[23] Wasyluk W Zwolak A. Metabolic alterations in sepsis. J Clin Med. (2021) 10(11):2412. doi: 10.3390/jcm10112412 34072402 PMC8197843

[24] Galley HF Lowes DA Allen L Cameron G Aucott LS Webster NR. Melatonin as a potential therapy for sepsis: a phase I dose escalation study and an ex vivo whole blood model under conditions of sepsis. J Pineal Res. (2014) 56:427–38. doi: 10.1111/jpi.12134 PMC4279949 24650045

[25] Lv C Huang L. Xenobiotic receptors in mediating the effect of sepsis on drug metabolism. Acta Pharm Sin B. (2020) 10:33–41. doi: 10.1016/j.apsb.2019.12.003 31993305 PMC6977532

[26] Ferreira FM Gomes SV Carvalho LCF de Alcantara AC da Cruz Castro ML Perucci LO. Potential of piperine for neuroprotection in sepsis-associated encephalopathy. Life Sci. (2024) 337:122353. doi: 10.1016/j.lfs.2023.122353 38104862

[27] Liang YD Bai WJ Li CG Xu LH Wei HX Pan H. Piperine suppresses pyroptosis and interleukin-1beta release upon ATP triggering and bacterial infection. Front Pharmacol. (2016) 7:390. doi: 10.3389/fphar.2016.00390 27812336 PMC5071324

[28] Li S Nguyen TT Ung TT Sah DK Park SY Lakshmanan VK. Piperine attenuates lithocholic acid-stimulated interleukin-8 by suppressing src/EGFR and reactive oxygen species in human colorectal cancer cells. Antioxid (Basel). (2022) 11(3), 530. doi: 10.3390/antiox11030530 PMC8944659 35326180

[29] Goto T Matsuzawa J Iemura S Natsume T Shibuya H. WDR26 is a new partner of Axin1 in the canonical Wnt signaling pathway. FEBS Lett. (2016) 590:1291–303. doi: 10.1002/1873-3468.12180 PMC5084729 27098453

[30] Jang J Song J Sim I Kwon YV Yoon Y. Wnt-Signaling Inhibitor Wnt-C59 Suppresses the Cytokine Upregulation in Multiple Organs of Lipopolysaccharide-Induced Endotoxemic Mice via Reducing the Interaction between beta-Catenin and NF-kappaB. Int J Mol Sci. (2021) 22(12):6249. doi: 10.3390/ijms22126249 34200709 PMC8230366

[31] Jang J Song J Lee H Sim I Kwon YV Jho EH. LGK974 suppresses lipopolysaccharide-induced endotoxemia in mice by modulating the crosstalk between the Wnt/beta-catenin and NF-kappaB pathways. Exp Mol Med. (2021) 53:407–21. doi: 10.1038/s12276-021-00577-z PMC8080716 33692475

[32] Calonghi N Boga C Telese D Bordoni S Sartor G Torsello C. Synthesis of 9-hydroxystearic acid derivatives and their antiproliferative activity on HT 29 cancer cells. Molecules. (2019) 24(20):3714. doi: 10.3390/molecules24203714 31619025 PMC6832665

[33] Micheletti G Calonghi N Farruggia G Strocchi E Palmacci V Telese D. Synthesis of novel structural hybrids between aza-heterocycles and azelaic acid moiety with a specific activity on osteosarcoma cells. Molecules. (2020) 25(2):404. doi: 10.3390/molecules25020404 31963693 PMC7024557

[34] Dongoran RA Lin TJ Byekyet A Tang SC Yang JH Liu CH. Determination of major endogenous FAHFAs in healthy human circulation: the correlations with several circulating cardiovascular-related biomarkers and anti-inflammatory effects on RAW 264.7 cells. Biomolecules. (2020) 10(12):1689. doi: 10.3390/biom10121689 33348748 PMC7766943

[35] Gadaleta RM Moschetta A. Metabolic Messengers: fibroblast growth factor 15/19. Nat Metab. (2019) 1:588–94. doi: 10.1038/s42255-019-0074-3 32694803

[36] Bourgonje AR Bolte LA Vranckx LLC Spekhorst LM Gacesa R Hu S. Long-term dietary patterns are reflected in the plasma inflammatory proteome of patients with inflammatory bowel disease. Nutrients. (2022) 14(12):2522. doi: 10.3390/nu14122522 35745254 PMC9228369

[37] Menoret A Buturla JA Xu MM Svedova J Kumar S Rathinam VAK. T cell-directed IL-17 production by lung granular gammadelta T cells is coordinated by a novel IL-2 and IL-1beta circuit. Mucosal Immunol. (2018) 11:1398–407. doi: 10.1038/s41385-018-0037-0 PMC6668340 29907868

[38] Yan D Ye S He Y Wang S Xiao Y Xiang X. Fatty acids and lipid mediators in inflammatory bowel disease: from mechanism to treatment. Front Immunol. (2023) 14:1286667. doi: 10.3389/fimmu.2023.1286667 37868958 PMC10585177

[39] Tanaka T Mori M Sekino M Higashijima U Takaki M Yamashita Y. Impact of plasma 5-hydroxyindoleacetic acid, a serotonin metabolite, on clinical outcome in septic shock, and its effect on vascular permeability. Sci Rep. (2021) 11:14146. doi: 10.1038/s41598-021-93649-z 34238999 PMC8266895

[40] Qiu J Wei L Su Y Tang Y Peng G Wu Y. Lipid metabolism disorder in cerebrospinal fluid related to Parkinson’s disease. Brain Sci. (2023) 13(8):1166. doi: 10.3390/brainsci13081166 37626522 PMC10452343

[41] Chaudhry H Zhou J Zhong Y Ali MM McGuire F Nagarkatti PS. Role of cytokines as a double-edged sword in sepsis. In Vivo. (2013) 27:669–84. PMC4378830 24292568

[42] Venet F Monneret G. Advances in the understanding and treatment of sepsis-induced immunosuppression. Nat Rev Nephrol. (2018) 14:121–37. doi: 10.1038/nrneph.2017.165 29225343

[43] Burgess S Thompson SG Collaboration CCG. Avoiding bias from weak instruments in Mendelian randomization studies. Int J Epidemiol. (2011) 40:755–64. doi: 10.1093/ije/dyr036 21414999

[44] Davies NM von Hinke Kessler Scholder S Farbmacher H Burgess S Windmeijer F Smith GD. The many weak instruments problem and Mendelian randomization. Stat Med. (2015) 34:454–68. doi: 10.1002/sim.6358 PMC4305205 25382280

