# Supplementary figures and images for: Dissecting the mediating role of inflammatory factors in the interaction between metabolites and sepsis: insights from bidirectional Mendelian randomization
Source: Front Endocrinol (Lausanne). 2024 Aug 14;15:1377755. doi: 10.3389/fendo.2024.1377755 (PMC11351091; doi:10.3389/fendo.2024.1377755)

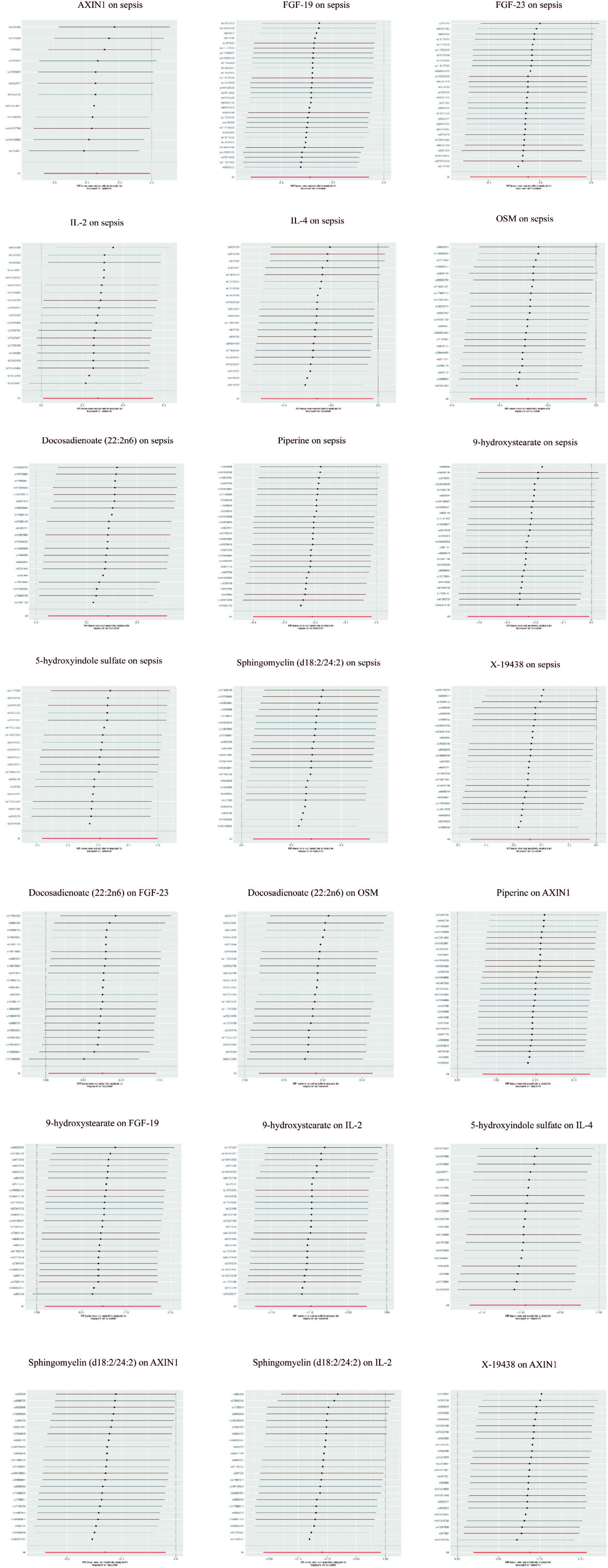

Supplement: Supplementary Figure 1 — Leave-one-out stability analysis. [file Image_1.jpeg]
