# Supplementary material for: Dissecting the mediating role of inflammatory factors in the interaction between metabolites and sepsis: insights from bidirectional Mendelian randomization
Source: Front Endocrinol (Lausanne). 2024 Aug 14;15:1377755. doi: 10.3389/fendo.2024.1377755 (PMC11351091; doi:10.3389/fendo.2024.1377755)
Supplement: Supplementary Table 8 — Ethics approval document. [file Table_8.pdf]

瑞金医院涉及人体科研伦理委员会委员会保密协议

Ruijin Hospital Ethics Committee
Confidential Agreement

作为瑞金医院伦理委员会成员，我将对我所审阅的“基于医院信息系统脓毒症快速精准诊断方法的建立与应用的多中心临床研究”有关资料以及伦理委员会会议的内容保密。

As an Ethics Committee member of Ruijin Hospital, I agree to hold the information of the protocol above and its relevant materials as a confidential manner.

姓名 Name	性别 Gender	职称 Title	委员会职务 Title in the EC	签名 Sign
瞿介明 Jieming Qu	男 Male	主任医师 Professor	主任 Chairman	
徐懿萍 Yiping Xu	女 Female	研究员 Research Fellow	副主任 Member	
毕宇芳 Yufang Bi	女 Female	主任医师 Professor	委员 Member	
卞留贯 Liuguan Bian	男 Male	主任医师 Professor	委员 Member	
丁健青 Jianqing Ding	男 Male	研究员 Research Fellow	委员 Member	缺席
董希会 Xihui Dong	男 Male	律师 Lawyer	委员 Member	
高卫益 Weiyi Gao	女 Female	副研究员 Research Associate	委员 Member	
李久辉 Jiuhui Li	男 Male	教授 Professor	委员 Member	
刘炳亚 Bingya Liu	男 Male	研究员 Research Fellow	委员 Member	
陆勇 Yong Lu	男 Male	主任医师 Professor	委员 Member	
夏振炜 Zhenwei Xia	男 Male	研究员 Research Fellow	委员 Member	
薛迪 Di Xue	女 Female	教授 Professor	委员 Member	
杨程德 Chengde Yang	男 Male	教授 Professor	委员 Member	
袁耀宗 Yaozong Yuan	男 Male	主任医师 Professor	委员 Member	
朱鼎良 Dingliang Zhu	男 Male	主任医师 Professor	委员 Member	
诸江 Jiang Zhu	男 Male	研究员 Research Fellow	委员 Member	缺席

地址：上海市瑞金二路 197 号
邮编：200025
电话：0086-021-64370045

No.197 Ruijin Er Road
Shanghai, P.R China,200025
Tel: 0086-021-64370045

Ruijin Hospital Ethics Committee
Shanghai JiaoTong University School of Medicine

涉及人体科研项目伦理委员会审批件
CLINICAL TRIAL ETHICS COMMITTEE APPROVAL FORM

伦理委员会编号
ETHICS COMMITTEE REFERENCE NUMBER:
（2021）临伦审第（59）号
(2021) Clinical Ethics Review No. 59

研究方案名称
STUDY TITLE:
基于医院信息系统脓毒症快速精准诊断方法的建立与应用的多中心临床研究
Establishment and application of a rapid and accurate diagnosis method for sepsis based on hospital information system: a multi-center clinical study

主要研究者/科室
PRINCIPAL INVESTIGATER /DEPARTMENT:
陈尔真 / 急诊科
Erzhen Chen, Emergency department

科研项目资助及编号
SPONSORED BY PROJECT / NO.
纵向课题（申康专项）
Longitudinal Project (Shen-Kang Special Program)

下面划[√]的研究相关文件已经审阅
The following items [√] have been reviewed in connection with the above study to be conducted by the above Investigator:

- [√] 研究方案 **Clinical Trial Protocol** 1.0/2020-12-20
- [√] 知情同意书 **Patient Information and Informed Consent Form** 1.0/2020-12-20
- [ ] 受试者招募广告 **Advertisement For Recruitment**
- [ ] 不良事件报告 **Serious Adverse Event**
- [ ] 其他 **Other**

审阅结果
And have been:

- [√] 无条件通过 **Approved**
- [ ] 有条件通过（请指出修改意见）**Conditionally Approved (identify item and specify modification below or in accompanying letter)**
- [ ] 拒绝（请指出原因）**Rejected（indentify item and specify reasons below or in accompanying letter）**

评价
Comments:
递交的研究方案及相关文件经伦理委员会审查，符合伦理规范，同意按递交方案进行临床研究。

上海交通大学医学院附属瑞金医院 伦理委员会

2021-3-4

Signature of Chairman **Date(DD/MMM/YYYY)**

Version 20210101

地址：上海市瑞金二路 197 号
邮编：200025
电话：0086-021-64370045

No.197 Ruijin Er Road
Shanghai, P.R China,200025
Tel: 0086-021-64370045
